# Effect of Germination on the Nutritional Properties, Phytic Acid Content, and Phytase Activity of Quinoa (*Chenopodium quinoa* Willd)

**DOI:** 10.3390/foods12020389

**Published:** 2023-01-13

**Authors:** Pedro Maldonado-Alvarado, Darío Javier Pavón-Vargas, Juan Abarca-Robles, Silvia Valencia-Chamorro, Claudia Monika Haros

**Affiliations:** 1 Department of Food Science and Biotechnology, Escuela Politécnica Nacional, Quito 17-01-2759, Ecuador; 2 Cereal Group, Instituto de Agroquímica y Tecnología de Alimentos IATA-CSIC, 46980 Valencia, Spain

**Keywords:** *Chenopodium quinoa*, germination, phytic acid, phytase activity

## Abstract

The aim of this study is to evaluate the effect of desaponification, soaking, germination, and refrigerated storage on the phytase activity, phytic acid content, and nutritional properties of three varieties of quinoa: white, red, and black. Desaponification and soaking reduced the number of minerals and the nutritional content. Germination of the seeds was carried out in the desaponified samples. The nutritional values, phytase activity, and phytic acid content of quinoa were measured after 6 h of soaking and then at 4 and 7 days during germination plus 7 days of refrigerated storage (4 °C). Germination increased the fibre and protein content as well as the iron, zinc, and calcium content. Germination significantly increased the phytase activity in all varieties and decreased the phytic acid content. The phytic acid content decreased during germination from 32 to 74%. Refrigerated storage had no significant effect on most of the factors studied. Germination boosts nutritional content and phytase activity while decreasing phytic acid content. Germination can be a simple method to reduce phytic acid in quinoa and may also improve the nutritional quality of this pseudocereal with the potential for use in functional foods and vegetarian diets.

## 1. Introduction

Quinoa (*Chenopodium quinoa* Willd), a pseudocereal, has been cultivated in the Andean region for thousands of years [1]. From a nutritional point of view, quinoa is a natural source of vegetable protein with high nutritional value due to its higher proportion of essential amino acids than in cereals [2,3]. Quinoa also has a high content of minerals, such as calcium, iron, zinc, and phosphorus [3,4]. In recent times, interest in this product has increased. It has become a popular raw material in the diet of vegetarians and people with intolerances or allergies to cereals. Researchers have shown particular interest in this pseudocereal as a potential ingredient in gluten-free food formulations [5,6].

Quinoa, however, has some anti-nutritional factors, especially saponins and phytic acid. Phytic acid can bind divalent minerals, making them unavailable for normal metabolism. As a consequence, phytic acid reduces the nutritional value of quinoa [7,8]. In humans, the consumption of 5 to 10 mg per day of phytic acid can cause a reduction in iron solubility of 50%, possibly leading to deficiencies of the mineral [9].

The phytase enzyme catalyses the hydrolysis of phytic acid, thus releasing inorganic phosphate from the seeds. The enzymatic activity of endogenous phytase in quinoa decreases with soaking, but, conversely, the enzymatic activity increases during germination due to the gradual dephosphorylation of the phytic acid complexes [10]. Germination not only reduces the amount of some anti-nutritional factors, such as phytic acid, but it also increases the amount of some nutrients, especially minerals and antioxidant compounds [7,11].

Germination is an important method to increase the nutritional and functional value of seeds. During germination, several enzymes are activated, improving protein digestibility and mineral bioavailability [12]. At present, there is a growing interest in the consumption of seed sprouts, alone or as a raw material for other products, because they have a higher nutritional value and are related to a healthier lifestyle [7].

The effect of germination has been studied in cereals and pseudocereals [13,14]. Recently, Pilco-Quesada et al. [14] studied the effects of germination and kilning on the phenolic compounds and nutritional properties of quinoa. They found that germination for 72 h resulted in a significant increase in the total content of phenolics and may improve the nutritional properties of the pseudocereal. However, even though there is some information about quinoa nutrients and anti-nutritional factors, previous studies have focused more on saponins [15], and there is no study focused on the phytic acid content and phytase activity of quinoa during germination and refrigerated storage. Thus, this research aimed at studying the effect of desaponification (saponin removal), soaking, germination, and refrigerated storage on the proximate composition, mineral content, phytic acid content, and phytase activity of three quinoa varieties.

## 2. Materials and Methods

### 2.1. Materials

#### 2.1.1. Raw Materials

Three varieties of quinoa were used for the experiments. White quinoa was supplied by INIAP (Instituto Nacional de Investigaciones Agropecuarias), Quito, Ecuador. Black quinoa and red quinoa harvested in Cotopaxi Province were supplied by Inca’s Treasure (Latacunga, Ecuador). The samples were stored at room temperature and in a dark environment to prevent light exposure.

#### 2.1.2. Desaponification, Germination, and Sampling Procedure

A wet method was used to remove the saponins in the seeds, which involved washing the quinoa with distilled water. The seeds were washed four times within a water–seed ratio of 2:1, with constant stirring at 100 rpm for 40 s. To verify the absence of saponins, a 3 g sample was shaken in a test tube together with 5 mL of distilled water. The formation or absence of foam after rinsing allowed us to determine if an additional wash should be carried out.

Quinoa seeds, previously desaponified, were disinfected with a Kilol^®^ solution (1 mL ascorbic acid, 0.475 mL citric acid, 0.47 mL lactic acid, water q.s.p. 100 mL–5 mL/L) (Chemie Ecuador, Guayaquil, Ecuador) prior to the germination process. The soaking and sprouting processes were carried out following the method described by D’Ambrosio et al. [12]. The seeds were soaked for 6 h in distilled water (water–seed ratio 2:1). Subsequently, the soaking water was drained, and the seeds were placed in trays, covered with humidified absorbent paper, inside a seed germinator (Mangelsdorf Germination Chamber, TE-406, Piracicaba, Brazil) for 7 days at a temperature of 20 °C and relative humidity (RH) of 100%.

The sampling was carried out 5 times during germination and storage. First, after soaking for 6 h (0.25 days), then at 4 and 7 days of germination in the chamber, and finally at 7 days of germination plus 4 and 7 days of refrigerated storage (days 11 and 14). In this sense, to evaluate the effect of refrigerated storage, after the 7th day of germination, sprouts of the three varieties were packaged in polyethylene terephthalate (PET) perforated boxes and stored in a refrigerator at a temperature of 4 °C and 75% RH for seven more days. Samples were taken at 7 plus 4 (11th) and 7 (14th) days of refrigerated storage for subsequent lyophilisation and storage as stated previously.

After 6 h of soaking (0.25 days), and at 4 and 7 days of germination, 35 g of each variety were sampled and frozen at −20 °C. The frozen samples were then freeze-dried in a lyophilizer (Stokes, 264 sq. ft., NC, USA), and then the samples were collected and stored in desiccators at room temperature until the chemical composition analysis was carried out.

**Scheme 1:**
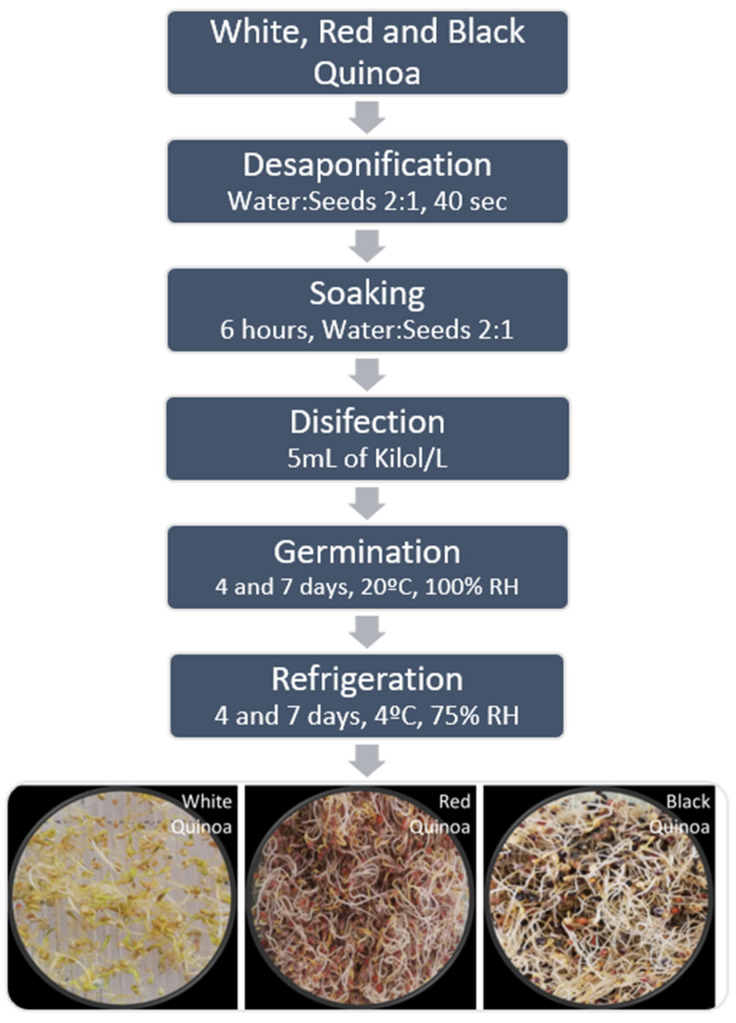
Desaponification, germination, and sampling procedure.

The whole procedure is shown in Scheme 1.

### 2.2. Methods

The chemical composition of the three varieties of quinoa (white, red, and black) was monitored before soaking (day 0), after soaking (0.25 days), at 4 and 7 days of germination, and at 4 and 7 days of refrigerated storage, to simulate the shelf-life of the sprouts.

#### 2.2.1. Proximate Analysis

The total lipid, fibre, ash, and moisture contents of the samples were determined according to AOAC official methods: 945.16, 985.29, 923.03, and 925.10, respectively [16]. Fibre was assessed using the test protocol adapted from the Megazyme^®^ Total Dietary Fibre Kit (Megazyme Ltd., Bray, Ireland). The protein content was analysed using the rapid nitrogen determination method according to

Dumas with a nitrogen and protein analyser (Elementar, Langenselbold, Germany). Carbohydrates were calculated using the other measurements by difference. All the analyses were performed three times.

#### 2.2.2. Mineral Content

The samples were analysed using atomic absorption spectrometry to determine the concentration of minerals. The metal analysis was completed using a flame absorption spectrometer at the Analysis of Soils, Plants and Water Service of the Institute of Agricultural Sciences, Madrid (Spain). to determine the calcium, zinc, and iron concentrations following the methodology proposed by Tazrart et al. [17]. Digestion of the samples was carried out using a microwave (MARS, Charlotte, NC, USA) with 4 mL of nitric acid (0.88 *m*/*v*) and 1 mL of hydrogen peroxide (33.3 *m*/*v*) at 180 °C. Nitric acid (≥99% purity) and hydrogen peroxide (≥99% purity) were obtained from Merck KGaA (Darmstadt, Germany). Six samples of each variety and time were run independently. Results were reported in milligrams (mg) per 100 g of sample.

#### 2.2.3. Phytic Acid Content

Quantification of the phytic acid content was measured according to the method proposed by Reason et al. [18] with a Phytic Acid Assay Kit by Megazymes^®^. Double enzymatic digestion was used to release inorganic phosphorus (Pi) from the samples. To produce a quantifiable value using UV-Vis spectroscopy, Pi was reacted with ammonium molybdate. The content of free and total inorganic phosphorus was measured as absorbance of molybdenum blue at a wavelength of 655 nm using a UV-Vis spectrometer (BMG Labtech, SPECTROstar Nano, Ortenberg, Germany). To quantify phytic acid in each sample a standard curve was prepared with dilutions of standard phosphorus. The content of free and total phosphorus was obtained from each sample, and the difference between these two values was the binding phosphorus. For each 0.282 g of binding phosphorus, there is 100 g of phytic acid.

#### 2.2.4. Phytase Activity

Potassium phytate was used as a source of phosphorus. Enzyme extracts of the samples were prepared following the methodology used by Luo et al. [19], which was modified by García-Mantrana et al. [20] for use on microplates. The absorbance of the reaction product was measured at 400 nm using a UV-Vis Spectrophotometer (BMG Labtech, SPECTROstar Nano, Ortenberg, Germany). A standard curve was prepared with a dipotassium phosphate stock solution. Potassium phytate (≥95% purity) and dipotassium phosphate (≥95% purity) were purchased from Sigma-Aldrich Co. (St. Louis, MO, USA). Phytase enzymatic activity was measured in phytase units (U), defined as the amount of enzyme capable of releasing 1 microgram (μg) of inorganic phosphorus per minute at a pH of 5.6 and a temperature of 50 °C.

### 2.3. Statistical Analysis

Statistical analyses of significant differences between the raw material and the desaponification and soaking processes were carried out for all the response variables. The results were expressed as mean value ± standard deviation (SD). The statistical analysis was performed with STATGRAPHICS Centurion XV (Statpoint Technologies Inc., Warrenton, VA, USA) software. One-way ANOVA was performed to evaluate the statistical significance of differences. At each storage moment, the difference of variables among the different quinoa varieties was tested with a post-hoc analysis using Fisher’s test (LSD) (*p* ≤ 0.05).

## 3. Results and Discussion

### 3.1. Chemical Composition of the Raw Material

The chemical composition of the raw quinoa samples is shown in Table 1. The moisture, lipid, protein, ash, and fibre contents are shown as percentages on a dry basis. There were significant differences (*p* ≤ 0.05) between the three varieties: white quinoa (WQ), red quinoa (RQ), and black quinoa (BQ) in moisture content, lipids, and ash.

In all varieties, the protein content ranged from 15.90% to 19.26%, which was in accordance with Valencia-Chamorro, who reported protein contents for quinoa between 8.0% and 22.0% [1,21]. Other authors have reported lower and higher crude protein values than those of the present study [7,22]. WQ protein content was significantly higher (18%) than that in the RQ and BQ samples. The ash content of the studied samples ranged from 2.27% to 4.27%, and the lipid content was around 7.5%, which is comparable to values in previous studies [14]. Although the fibre content values for the black and white varieties did not show significant differences and were in the range found in the literature, the fibre content value in the red variety was around 19.55%, which was much higher than the range reported for crude fibre in quinoa (2.5–3.9%) [1,14]. The reason for the high variability in the proximate analysis among the quinoa samples may be due to the variety of each seed. It is known that quinoa varieties and the growing environment can affect protein content [2,23].

As can be seen in Table 1, the mineral content of samples in this investigation showed significant differences (*p* ≤ 0.05) for calcium and zinc. The calcium content in RQ and BQ was 76.76 and 74.72 mg/100 g, respectively, which was significantly higher (14.3%) than the calcium content in WQ (64.88 mg/100 g). Conversely, the zinc content in WQ was significantly higher (54.3%) than the zinc content in the RQ and BQ varieties. The mineral content values obtained in this study were similar to those described in the literature [4,24].

It is important to note that the presented values for the chemical composition correspond to quinoa seeds before desaponification and without scarification. The results obtained do not consider losses from the desaponification process, e.g., Repo-Carrasco-Valencia and Astuhuaman Serna [22] reported losses between 0.71 and 2.95 g/100 g of protein after desaponification of quinoa seeds.

### 3.2. Phytic Acid Content and Phytase Activity of the Raw Material

The phytic acid content of BQ (1.22 g/100 g) was significantly higher than that of WQ (1.07 g/100 g) and RQ (1.03 g/100 g) (Table 1). It has been reported that, for different varieties of quinoa, the average value of the phytic acid content is 1.18 g/100 g [1]. Higher values up to 1.94 g/100 g [25] and lower values (0.97 g/100 g) have been reported for the white quinoa variety [7,25]. The phytic acid content in quinoa, in general, presents higher values than those of other cereals and seeds [10]. In quinoa, phytic acid is not only present in the outer layers of the seed, as in other cereals, but is also evenly distributed in the endosperm [26]. A high phytic acid content can have a negative effect on nutrition and diet [9]. A low phytic acid content is preferred.

Phytase activity was measured in phytase units (U). As can be seen in Table 1, RQ presented the lowest phytase activity value of 37.01 ± 0.34 U, BQ showed a value of 40.01 ± 0.39 U, and the highest phytase activity was found in WQ at a value of 41.56 ± 0.99 U. There were significant differences (*p* ≤ 0.05) between the three varieties.

### 3.3. Effect of Germination and Refrigerated Storage on the Chemical Composition

#### 3.3.1. Protein Content

**Figure 1:**
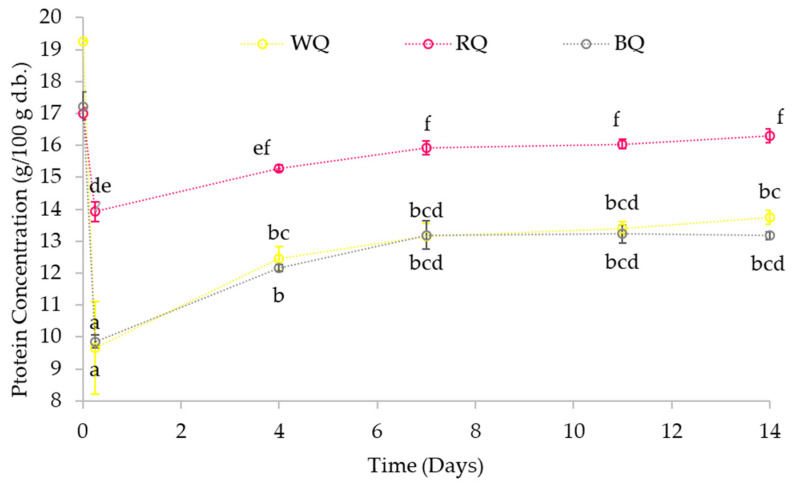
Protein content variation for white (WQ), red (RQ), and black (BQ) quinoa, during soaking (0.25 days), germination (4 and 7 days at 20 °C and 100% RH), and refrigerated storage (11 and 14 days at 4 °C and 75% RH). Error bars indicate standard deviation (*n* = 3). Results with different letters are significantly different at *p* ≤ 0.05.

The protein content of the studied samples decreased after soaking but increased during germination. Figure 1 shows the change in protein content for the three varieties of quinoa during soaking, germination, and refrigerated storage. As can be seen, after the desaponification and soaking processes (0.25 days), the total protein content in all the varieties had reduced from the initial value. WQ showed the most significant reduction (59%) in protein compared to the raw materials, while in RQ and BQ, the protein concentration was reduced by 40% and 14%, respectively.

According to Repo-Carrasco-Valencia and Astuhuaman Serna [22], the desaponification process causes a decrease in protein content. They reported a reduction of up to 7% in the protein content of quinoa after desaponification. The soaking process was not considered in their study, which could explain the more significant percentage of protein reduction in the present study. The main protein present in quinoa is globulin [27], which, since it is soluble in water, could be leached as a result of the soaking processes together with other soluble proteins and molecules.

Germination had a significant effect (*p* ≤ 0.05) on the protein content of quinoa sprouts compared to the values after soaking. There was an increasing trend in the three varieties of quinoa during germination. On day 4, WQ and BQ increased their protein concentration by 1.29 and 1.23 times, respectively, compared to the protein concentration after the desaponification and soaking processes. However, RQ required a longer germination time (7 days) to achieve a significant difference compared to the protein concentration after soaking. An increase in protein content can be also attributed to weight loss during respiration due to the carbohydrates and lipids consumption pathways, as absolute protein content cannot change [14]. In this study, a reduction in the lipid and carbohydrate content was observed for all the samples. The quinoa variety was also a factor that affected protein concentration, and there were significant differences between the varieties. At the end of the study, RQ reached a significantly higher level of protein content than WQ and BQ. In contrast to the results found, Chaparro et al. [28] showed that there were no significant differences for the ‘Valle del Cauca’ quinoa variety after the germination process.

Following the plus 7 days of refrigerated storage (4 °C and 75% RH) after the germination process, no differences were shown in any sample until day 14. The protein concentration in the sprouts remained at the levels reached during germination.

#### 3.3.2. Ash Content

**Figure 2:**
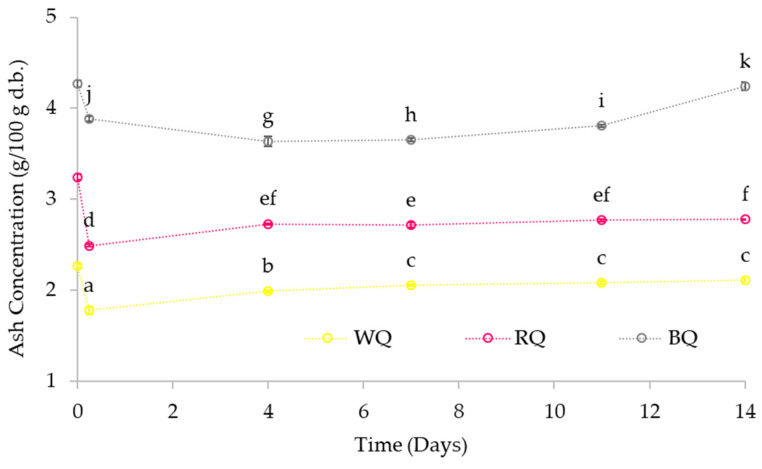
Ash content variation for white (WQ), red (RQ), and black (BQ) quinoa during soaking (0.25 days), germination (4 and 7 days at 20 °C and 100% RH), and refrigerated storage (11 and 14 days at 4 °C and 75% RH). Error bars indicate standard deviation (*n* = 3). Results with different letters are significantly different at *p* ≤ 0.05.

The variation in ash content of the quinoa samples during soaking, germination, and storage is given in Figure 2. Ash contents of the quinoa sprouts at the end of the storage changed between 2.11 and 4.24%. The germination process increased the values between 9 and 18% relative to the values after soaking (1.78–3.88%). The final values were close to those reported for the initial raw material (0 days), which were between 2.27 and 4.27%.

Germination significantly affected (*p* ≤ 0.05) the ash content present in the sprouts after soaking. In the white variety, after the desaponification and soaking processes, the ash content decreased by 22% compared to the raw material, and then, the ash content significantly increased (*p* ≤ 0.05) until day 7 of germination. The same behaviour was observed in the red variety but without a statistical difference between the fourth and seventh days of germination. In these two varieties, there were no changes in the ash content during refrigerated storage, and the values reached on day 7 of germination were maintained. For the black variety (BQ), the effect of germination and refrigerated storage had a different trend than observed in the previous varieties. In BQ, the ash content present in the raw material decreased by 9% after soaking; subsequently, the value progressively increased during germination and then during refrigerated storage until day 14.

The initial reduction of ash content during soaking can be explained by the loss of minerals due to lixiviation [14]. Meanwhile, the increase in ash content during germination may be related to the conversion of carbohydrates to carbon dioxide during respiration, therefore, increasing the percentage of the ash on a dry basis. These data corroborate those found in other studies for quinoa and quinoa flour, which showed significant increases in ash content after germination of 33% and 46%, respectively [7,29]. Nevertheless, another study indicated a reduction in ash content in sprouted quinoa compared to the raw material, which was explained by the use of minerals as coenzymes during the bioconversion of carbohydrates [14].

There was an increase in ash content in the black variety due to refrigeration. Refrigerated storage should preserve the original composition, as in the case of ash. Metabolic activity during refrigeration, including respiration, consumes water and other products, such as carbohydrates or lipids. This can be a form in which the ash increases in percentage by reducing the content of other components. Furthermore, in this study, the relative humidity in refrigeration was lower than in the germination process. Thus, the water of the product could be lost more easily in the sprouts and, therefore, a higher ash content during refrigerated storage could be achieved. Quinoa has high genetic variability, and the different increments in the amount of ash present in the samples during germination and storage could be linked to it [30].

#### 3.3.3. Fibre Content

**Figure 3:**
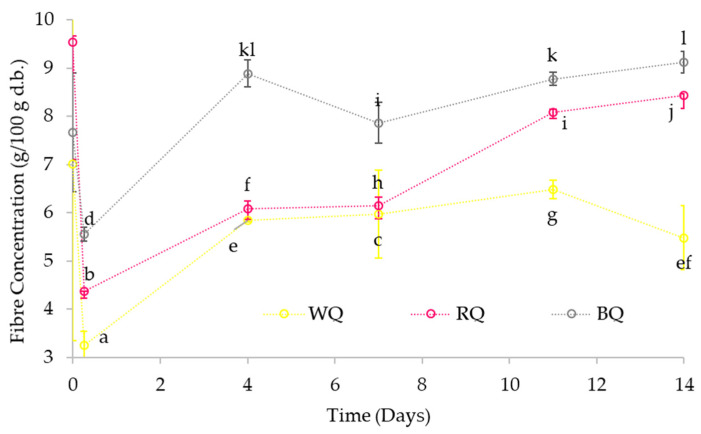
Fibre content variation for white (WQ), red (RQ) and black (BQ) quinoa during soaking (0.25 days), germination (4 and 7 days at 20 °C and 100% RH), and refrigerated storage (11 and 14 days at 4 °C and 75% RH). Error bars indicate standard deviation (*n* = 3). Results with different letters are significantly different at *p* ≤ 0.05.

Figure 3 shows the effects of the treatments on the fibre content of the three quinoa varieties. Desaponification and soaking caused a significant reduction in fibre content of between 28 and 78%. The loss of the perianth during desaponification and soaking may explain this decrease. The perianth in the quinoa seeds contains insoluble fibre that can be lost during soaking [31]. The fibre content values after soaking, as well as during germination and refrigerated storage, were within the values found for raw and germinated quinoa [1,14].

Germination significantly increased (*p* ≤ 0.05) the fibre content values for the three varieties compared to values after soaking (0.25 days). The synthesis of fibre within the structures, such as the hypocotyl and the radicle, of quinoa seeds during germination could explain this increase [32]. However, there was no significant change between the values of the 4th day (between 5.84 and 8.89%) and the 14th day (between 5.48 and 9.13%) after refrigerated storage, although there were differences between the three varieties. Similar results were described for quinoa after 3 days of germination, where there was no significant effect of the germination process on the crude fibre content [14].

#### 3.3.4. Lipid and Carbohydrate Content

**Figure 4:**
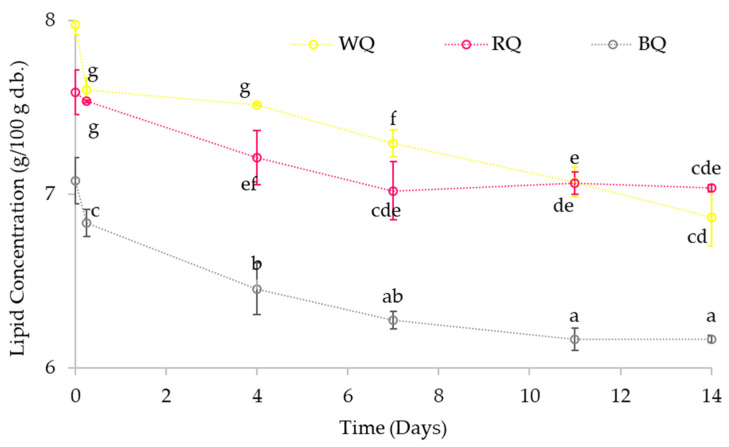
Lipid content variation for white (WQ), red (RQ), and black (BQ) quinoa during soaking (0.25 days), germination (4 and 7 days at 20 °C and 100% RH), and refrigerated storage (11 and 14 days at 4 °C and 75% RH). Error bars indicate standard deviation (*n* = 3). Results with different letters are significantly different at *p* ≤ 0.05.

Total lipid content in the three varieties showed a significant difference between the sampling days. A decreasing trend during germination and refrigerated storage was observed (Figure 4). During the autotrophic development of quinoa, seeds do not perform photosynthesis, and lipids are most likely a source of energy during germination [32]. As for carbohydrates, they also decrease as the germination process progresses, as carbohydrates are used as an energy source during the germination process (Table 2). The seeds use carbohydrates and lipids for biochemical activities due to germination [33]. This reduction in lipid and carbohydrate content was also observed in a study by Pilco-Quesada et al. [14] on germinated quinoa seeds.

#### 3.3.5. Calcium

Germination brought about a great increase in the calcium content of the samples. The increase ranged from 25% to 27% in the three varieties compared to the material after desaponification and soaking, and it increased significantly for the white and black varieties, by 17% and 26%, respectively, compared to the values of the initial raw material (Table 2). Calcium content in the samples at the end of the germination period ranged between 75.9 and 93.5% mg/100 g (d.b.). Quinoa sprouts have a relatively higher content of calcium compared to raw quinoa and other cereals [1].

Similar results for the influence of quinoa seed germination on calcium concentration were obtained by Chaparro et al. [28] who described an increase of 25% in quinoa after two days of germination. Germination, in general, has been shown to promote the availability of minerals in cereals [33]. Contrary to this, refrigerated storage did not affect the calcium content in any of the germinated samples.

The loss of the perianth during saponification, together with the leaching resulting from soaking, are two determinant factors in the initial reduction of calcium content during these processes [34]. Although, the increase in calcium content during germination may be related to the phytic acid content and phytase activity. As germination progresses, the chelation of metals becomes reduced. Therefore, a higher content of calcium, iron, and zinc is present in the samples.

#### 3.3.6. Zinc

After the desaponification and soaking processes, WQ presented the highest increase in zinc content with respect to raw materials by 5%, while in RQ, the zinc content increased by 1.8%, and in BQ, the concentration of zinc decreased by 0.8% (Table 2).

The germination process produced a significant increase (*p* ≤ 0.05) in the zinc content after 4 days of germination in all the varieties, which increased by 10, 14, and 45% for the WQ, BQ, and RQ, respectively, when compared to the zinc contents after desaponification and soaking. Furthermore, the zinc concentrations after seven days of germination were higher than in the raw materials by 15, 13, and 43% for WQ, BQ, and RQ, respectively. Although refrigerated storage did not influence the zinc content in WQ and BQ after germination, it brought a significant decrease (*p* ≤ 0.05), by 25% at the 7 days plus 7 days of refrigerated storage, in the red variety.

Luo et al. [35] showed an increase in the availability of zinc for wheat and rice seeds, by 80 and 87%, respectively, after germination. Nevertheless, in soybeans, zinc availability decreased by 73%. Zinc participates in the hydrolysis of reserve substances in the seeds, and this could reduce its content during refrigeration, for example. On the other hand, during germination, the increase in zinc content could be explained by the physiology of the plant itself [36].

#### 3.3.7. Iron

All the varieties had similar iron contents, ranging from 4.45 to 4.81 mg/100 g (d.b.) in the raw material. After the desaponification and soaking processes, RQ showed a decrease of 37% in iron content. Meanwhile, in the WQ and BQ varieties, the reduction in iron concentration was 10 and 5.53%, respectively (Table 2). Iron concentration for the WQ and BQ varieties increased significantly (*p* ≤ 0.05) with germination until the seventh day. Nevertheless, the RQ variety did show significant differences (15%) in iron concentration due to germination. Chaparro et al. [28] reported a significant increase (11.40%) after one day of germination in the ‘Valle del Cauca’ quinoa variety. Moreover, the germination process significantly increased the amount of iron in other cereals, with a 2.45% and 4.24% increase for rice and wheat, respectively [35]. Refrigerated storage, however, did not have any effect on the iron content of the quinoa sprouts as the values remained similar to those found on day 7 of germination in all varieties.

Since phytic acid is one of the factors affecting mineral bioavailability, its reduction during germination would enhance the mineral content [33]. Thus, germination generally increased the availability of minerals in the quinoa samples. Another factor that influenced the mineral content was leaching during soaking. Not only do the germination and leaching processes influence the concentration of calcium, zinc, and iron, but also the variety. The nutritional composition of quinoa greatly varies according to some factors, including the genotype of quinoa [30].

### 3.4. Effect of Germination and Refrigerated Storage on the Phytic Acid Content and Phytase Activity

#### 3.4.1. Effect on the Phytic Acid Content

**Figure 5:**
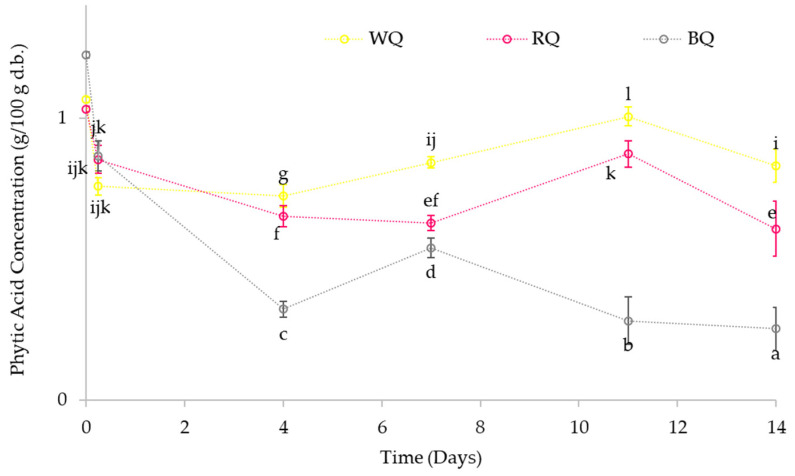
Effect of germination and refrigerated storage on the phytic acid content in three varieties of quinoa: white (WQ), red (RQ), and black (BQ). Error bars indicate standard deviation (*n* = 3). Results with different letters are significantly different at *p* ≤ 0.05.

The effect of germination and refrigerated storage on phytic acid content for the three quinoa varieties is presented in Figure 5. A statistically significant reduction in phytic acid was observed due to desaponification and soaking. A second significant reduction was observed for all treatments at the beginning of germination until day 4. Afterwards, the content of phytic acid on the last day of refrigerated storage was also significantly decreased as compared to day 11 of refrigeration and the values in germination. The decrease in phytic acid content in pulses and cereals during germination has been frequently reported in the literature [10]. The phytate reduction after the whole process was 22%, 41%, and 79% in WQ, RQ, and BQ, respectively. The reduction was very effective in the BQ where the molar ratio of Ca and Zn were lower than the threshold value for inhibition of mineral bioavailability in humans [37].

The phytic acid content in the three quinoa varieties after the soaking process decreased. This behaviour could be inferred by the leaching during the soaking of the seeds [10]. During germination on day 4, WQ and BQ presented phytic acid values of 0.72 and 0.32 g/100 g (d.b.), respectively; these values were lower (32.7% and 73.7%, respectively) than the content of phytic acid reported for the raw materials and seeds after soaking. However, RQ took three more days of germination to reach the minimum value, with a phytic acid content value of 0.63 g/100 g (d.b.), which was 67.9% lower than the raw material. The most significant reduction in phytic acid was found in black quinoa with a decrease of 73.54% compared to the initial material.

It has been shown that the soaking and germination processes had a significant effect on the decrease of phytic acid content in beans [38]. The positive effect of increasing the content of some minerals may be also attributed to the decrease in phytic acid content as a result of germination. The phytate complex is known as a chelating agent that reduces mineral contents [19]. It has been reported that during germination, cereals and legumes produce synthesis and activation of endogenous phytases which reduces phytate levels [39].

#### 3.4.2. Effect on Phytase Activity

**Figure 6:**
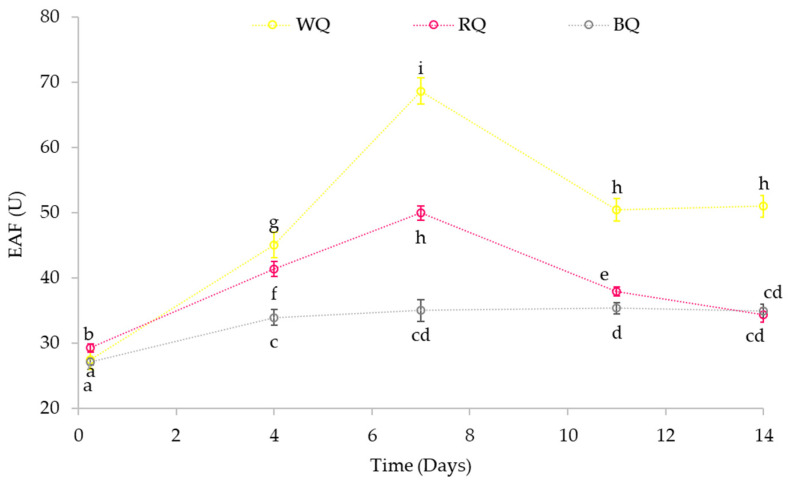
Effect of germination and refrigerated storage on phytase enzymatic activity in three varieties of quinoa: white (WQ), red (RQ), and black (BQ). Error bars indicate standard deviation (*n* = 3). Results with different letters are significantly different at *p* ≤ 0.05.

Figure 6 shows the effect of germination and refrigerated storage on the phytase activity of the three quinoa varieties. The phytase activity in the quinoa samples was significantly increased as a result of germination. The percentage increase on day 7 was found to be between 64.10 and 149.00% compared to the reduced values after soaking. Accordingly, during germination, the phytate levels decreased while the phytase activity increased. During soaking (0.25 days), the phytase activity decreased compared to the initial material by 31.97, 33.85, and 37.01% for BQ, RQ, and WQ, respectively. The phytase activity in seeds was reduced during soaking, and this decrease could be explained by phytase leaching [10].

In the WQ and RQ varieties, an increasing trend in phytase activity was observed during germination until day seven, whereas, for the black variety, the increase was shown until the fourth day. WQ had the highest value for phytase activity of the three varieties by the 7th day of storage, with a value of 68.65 U. According to Sung et al. [40], an increase in phytase activity can be explained by the synthesis of new enzymes during the germination process.

Refrigeration had a negative effect on the phytase activity. The phytase activity was reduced during refrigerated storage until day 14, which can possibly be explained by the low temperature (4 °C), which was far from optimal for this enzyme [41]. The decrease in the white variety was up to 50.98 U at 7 days of refrigerated storage. For the RQ and BQ, the enzymatic activity had a lower value, and no significant differences were found between the two samples after 7 days of refrigerated storage. The reduction occurs from day 7 of germination. However, there were no significant differences between days 11 and 14 of refrigerated storage. According to Spier et al. [41], who studied the effect of refrigerated storage on phytase activity, a temperature reduction up to 4 °C causes a decrease in phytase activity in a range that fluctuates between 36.2 and 40.0%. This reduction may also be due to the degradation of phytase by the protease enzymes [42].

## 4. Conclusions

In the present work, it was found that the white quinoa variety had the highest protein, lipid, and zinc content, while the red variety had the highest fibre content. In general, desaponification and soaking reduced the nutritional content of the samples in the three varieties. However, germination boosts nutritional content and phytase activity while decreasing phytic acid content. It is concluded that the germination process can be a simple method to increase phytase activity and decrease the phytic acid content, as well as improve the nutritional values of quinoa. In this study, it was found that germination caused an increase in fibre and protein content, along with an increase in the percentage of calcium, zinc, and iron. After 7 days of germination, the most increased calcium and iron contents were found in the white variety. Meanwhile, refrigerated storage did not produce any significant change in the nutritional content. The most increased enzymatic activity was found in white quinoa after 7 days of germination, while the largest reduction in phytic acid content was presented in the black variety after refrigeration. A maximum activity value for the phytase enzymes was observed on day 7 of germination, and then a decrease was observed during refrigerated storage. The germination process has the potential to be an easy method to create a functional product for food production and vegetarian diets.

## Figures and Tables

**Table 1:** Chemical composition of the raw quinoa samples (d.b.).

	White Quinoa	Red Quinoa	Black Quinoa
Moisture (%)	13.26 ± 0.04 ^c^	12.44 ± 0.01 ^a^	12.55 ± 0.04 ^b^
Lipids (%)	7.92 ± 0.06 ^c^	7.56 ± 0.13 ^b^	7.02 ± 0.13 ^a^
Crude protein (%)	19.26 ± 0.95 ^b^	15.90 ± 0.12 ^a^	16.06 ± 0.04 ^a^
Ash (%)	2.27 ± 0.02 ^a^	3.25 ± 0.04 ^b^	4.27 ± 0.08 ^c^
Fibre (%)	7.02 ± 3.68 ^a^	19.55 ± 2.43 ^b^	7.68 ± 1.24 ^a^
Carbohydrates (%)	50.27	41.3	53.42
Calcium (Ca) (mg/100 g) *	64.88 ± 0.84 ^a^	76.76 ± 4.87 ^b^	74.72 ± 8.49 ^b^
Zinc (Zn) (mg/100 g) *	3.83 ± 0.08 ^b^	1.77 ± 0.16 ^a^	1.73 ± 0.25 ^a^
Iron (Fe) (mg/100 g) *	4.45 ± 0.36 ^a^	4.81 ± 0.25 ^a^	4.68 ± 0.48 ^a^
Phytic acid (g/100 g)	1.07 ± 0.01 ^b^	1.03 ± 0.03 ^a^	1.22 ± 0.01 ^c^
Phytase activity (U)	41.56 ± 0.99 ^c^	37.01 ± 0.34 ^a^	40.01 ± 0.39 ^b^

Mean ± SD ( *n* = 3, *n* = 6). Results with different superscript letters within the same row are significantly different at *p* ≤ 0.05. The “*” means that the umber of determination of Ca, Fe and Zn were 6. [Mean ± SD, *n* = 3, (* *n* = 6)].

**Table 2:** Carbohydrate and mineral content variation for white (WQ), red (RQ), and black (BQ) quinoa during soaking (0.25 days), germination (4 and 7 days at 20 °C and 100% RH), and refrigerated storage (11 and 14 days at 4 °C and 75% RH).

Component	Units	Quinoa	Desaponification and Soaking 0.25 Days	Germination 4 Days	Germination 7 Days	Refrigeration Storage 4 Days	Refrigeration Storage 7 Days
Carbohydrates*	g/100 g d.b.	WQ	71.67	66.04	65.29	64.80	64.80
		RQ	63.96	60.96	60.46	58.55	56.60
		BQ	67.47	61.24	61.95	60.74	60.14
Ca	mg/100 g d.b.	WQ	60.5 ± 2.4 ^bcd^	66.2 ± 4.4 ^de^	75.6 ± 5.4 ^f^	74.4 ± 7.0 ^f^	71.3 ± 3.9 ^ef^
		RQ	51.6 ± 0.3 ^a^	56.8 ± 1.1 ^ab^	64.6 ± 1.4 ^cde^	57.5 ± 4.0 ^abc^	63.2 ± 6.5 ^bcd^
		BQ	73.9 ± 5.9 ^f^	84.4 ± 4.1 ^g^	94.1 ± 7.3 ^hi^	91.5 ± 7.6 ^gh^	97.0 ± 8.2 ^i^
Zn	mg/100 g d.b.	WQ	4.0 ± 0.1 ^h^	4.4 ± 0.2 ^i^	4.6 ± 0.1 ^i^	4.7 ± 0.0 ^i^	4.7 ± 0.3 ^i^
		RQ	1.8 ± 0.0 ^ab^	2.6 ± 0.6 ^fg^	2.8 ± 0.3 ^g^	2.1 ± 0.1 ^cd^	2.2 ± 0.2 ^cde^
		BQ	1.7 ^a^	2.0 ^abc^	2.1 ^bcd^	2.2 ^de^	2.4 ^ef^
Fe	mg/100 g d.b.	WQ	4.0 ± 0.4 ^abde^	nd	5.2 ± 0.5 ^efg^	4.9 ± 0.1 ^cdfg^	5.0 ± 0.2 ^defg^
		RQ	3.0 ± 0.1 ^a^	nd	3.4 ± 0.1 ^ab^	3.6 ± 0.1 ^abc^	3.3 ± 0.5 ^ab^
		BQ	4.4 ± 0.4 ^bdf^	nd	5.7 ± 0.9 ^fg^	5.8 ± 0.3 ^g^	4.9 ± 0.3 ^bdfg^

Mean ± SD, *n* = 6. Results with different superscript letters within the same row are significantly different at *p* ≤ 0.05. * Carbohydrates were calculated based on the other component measurements by difference. n.d.: No determined.

## Data Availability

Data is contained within the article.
